# Pancreatic Pseudocyst Pleural Fistula in Gallstone Pancreatitis

**DOI:** 10.1155/2016/4269424

**Published:** 2016-05-05

**Authors:** Sala Abdalla, Ioannis Nikolopoulos, Rajab Kerwat

**Affiliations:** Department of General Surgery, Queen Elizabeth Hospital, Stadium Road, Woolwich, London SE18 4QH, UK

## Abstract

Extra-abdominal complications of pancreatitis such as pancreaticopleural fistulae are rare. A pancreaticopleural fistula occurs when inflammation of the pancreas and pancreatic ductal disruption lead to leakage of secretions through a fistulous tract into the thorax. The underlying aetiology in the majority of cases is alcohol-induced chronic pancreatitis. The diagnosis is often delayed given that the majority of patients present with pulmonary symptoms and frequently have large, persistent pleural effusions. The diagnosis is confirmed through imaging and the detection of significantly elevated amylase levels in the pleural exudate. Treatment options include somatostatin analogues, thoracocentesis, endoscopic retrograde cholangiopancreatography (ERCP) with pancreatic duct stenting, and surgery. The authors present a case of pancreatic pseudocyst pleural fistula in a woman with gallstone pancreatitis presenting with recurrent pneumonias and bilateral pleural effusions.

## 1. Introduction

Pancreaticopleural fistula (PPF) is one of the rarest complications of acute and chronic pancreatitis. It is characterised by the presence of amylase-rich pleural fluid resulting from an established fistulous communication between the pancreas and/or pancreatic pseudocyst and the pleural cavity [1, 2]. Leakage of secretions from the pancreas or ruptured pancreatic pseudocyst may be fistulated into the thorax directly through the diaphragm or through the aortic and oesophageal diaphragmatic hiatuses. Involvement of the left pleural space is more common and patients typically present with pulmonary symptoms such as dyspnoea, cough, and chest pain [1]. The diagnosis is often delayed and many patients undergo extensive pulmonary investigations before the final diagnosis is reached. For this reason, PPF must be suspected in individuals with recurrent pulmonary effusions and concomitant history of pancreatitis. Here we present a case of PPF in a woman with gallstone pancreatitis presenting with recurrent pneumonias and bilateral pleural effusions.

## 2. Case Report 

A 65-year-old woman presented with a short history of shortness of breath (SOB), generalised abdominal pain, and poor oral intake. She had a background of type 2 diabetes mellitus, hypercholestrolaemia, pancreatitis, and recurrent pulmonary effusions. She did not drink alcohol and was an ex-smoker of 20 cigarettes per day, having stopped 32 years previously. She had had previous indirect asbestos exposure in that her husband builder worked directly with asbestos and she handled his clothes. She was otherwise independent.

Six months previously she was admitted with acute, severe necrotizing pancreatitis of unclear aetiology. Ultrasound (US) scanning during that admission showed no gallstones and computed tomography (CT) scanning confirmed the presence of necrosis in the body and tail of the pancreas. Fasting lipid and calcium profiles at the time were within normal limits. The cause of the pancreatitis was suspected to be the ezetimibe she was taking for hypercholesterolaemia and this was stopped.

Over the next six months she was admitted twice more to hospital with large left-sided pleural effusions which were managed with tube thoracostomy. During both admissions which were two months apart, the pleural fluid amylase was 17409 iu/L and 16783 iu/L with no evidence of malignant cells or pus cells. Magnetic resonance cholangiopancreatography (MRCP) and CT scanning during the admissions showed no evidence of gallstones nor pancreas divisum but revealed cysts in the body and tail of pancreas measuring 5.3 and 6.2 cm. One month later, a follow-up US scan to assess the progress of the pancreatic cysts showed that they decreased to 3.7 and 2.4 cm, respectively. However, on this occasion, the US scan detected several small gallstones with no evidence of biliary tree dilatation.

**Figure 1:**
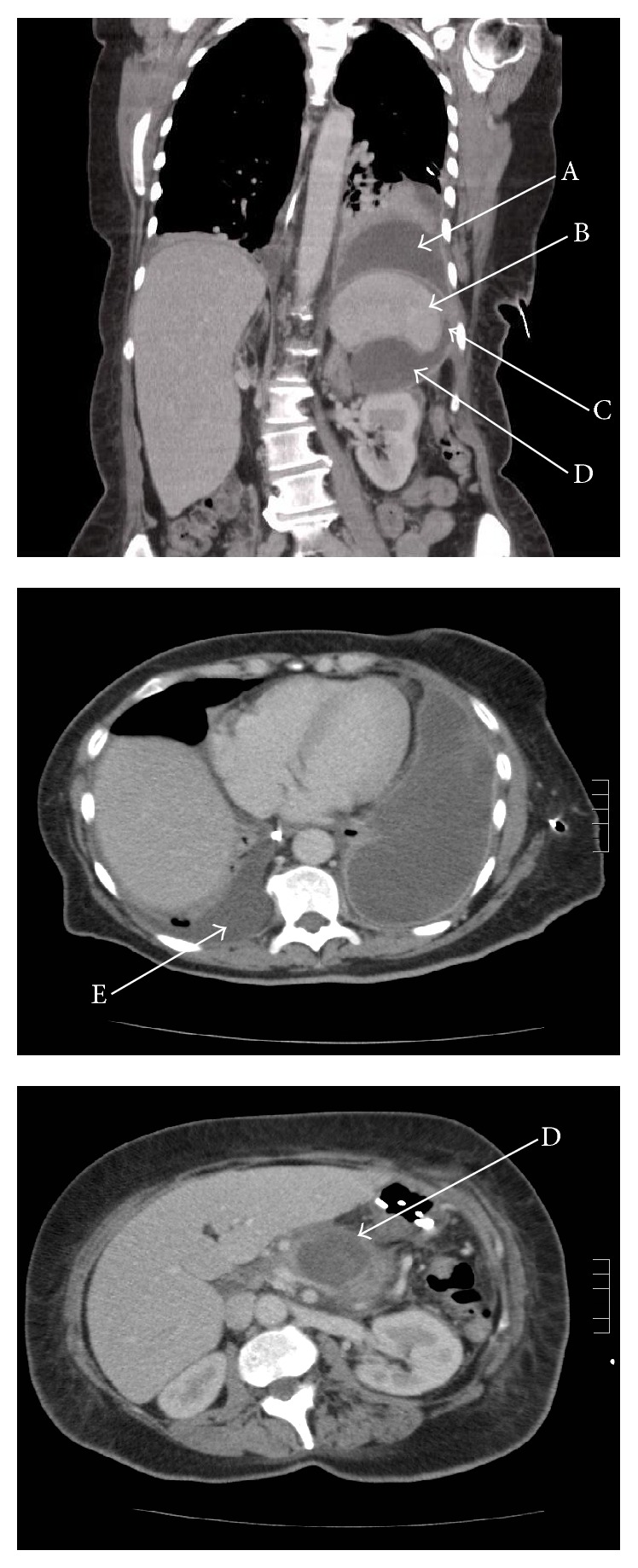
CT scan of the chest and abdomen demonstrating bilateral pleural effusions, the largest being in the left pleural cavity which is communicating directly with a pancreatic pseudocyst confirming the diagnosis of pancreatic pseudocyst-pleural fistula (PP-PF). Arrow A shows the position of the left pleural effusion communicating through fistula C with a large pancreatic pseudocyst D at the posterolateral aspect of the spleen B. Arrow E demonstrates the smaller right-sided pleural effusion.

During this presentation with SOB and abdominal pain, six months following her initial admission with acute pancreatitis, she was found to have a serum amylase of 1056 and bilateral pleural effusions. She was no longer on ezetimibe. Computed tomography scanning of the chest, abdomen, and pelvis revealed bilateral pleural effusions and a pancreatic pseudocyst that was communicating with the left pleural space suggestive of a pancreatic pseudocyst-pleural fistula (PP-PF) (Figure 1). The left pleural effusion was treated with thoracostomy, and the right was managed conservatively. She was commenced on octreotide to reduce the pancreatic exocrine secretions, a proton pump inhibitor, and nasojejunal feeding. Thereafter she was managed in a specialist hepatopancreaticobiliary unit where she underwent endoscopic cystogastrostomy. One month following the procedure, a CT scan confirmed resolution of the pancreatic pseudocyst. A month after this, she underwent elective laparoscopic cholecystectomy given the eventual detection of gallstones on her most recent transabdominal and endoscopic US scans. She remains well with no further occurrence of pancreatitis and no radiological evidence of recurrence of the pancreatic pseudocyst or the pleural effusions.

## 3. Discussion

Pancreaticopleural fistulas (PPFs) occur in 0.4% of patients with pancreatitis and in 6–14% of patients with pancreatic pseudocysts, although they can also present following surgical pancreatic resections, percutaneous drainage of a pseudocyst, and abdominal trauma [1–3]. Eighty percent of cases are associated with alcohol-induced chronic pancreatitis, with gallstone aetiology being much less common [4, 5]. In the case presented, the patient was initially thought to have drug-induced pancreatitis secondary to her long-term antilipid drug: ezetimibe. However, she went on to have further attacks of pancreatitis after cessation of ezetimibe and was later on found to have small gallstones. It is likely that her underlying aetiology was gallstones that were missed in her initial scans.

Leakage of pancreatic secretions can cause significant morbidity due to malnutrition and sepsis. When the secretions leak posteriorly, they track cranially into the pleura forming PPFs. Involvement of the left pleural space is by far the commonest, accounting for 76% of cases, and pleural effusions tend to be recurrent despite repeated thoracocentesis [6, 7]. Involvement of both pleural spaces is less common and has been reported in 14% of patients with pancreaticopleural fistulas. The subject of this case report had presented mainly with unilateral left-sided pleural effusions, although in her last presentation when the diagnosis of PPF was made, she had bilateral pleural effusions. Patients with PPF present predominantly with pleura-pulmonary symptoms rather than abdominal symptoms. In some cases, a PPF may be the first presentation of pancreatic disease as described in a study by Fulcher and colleagues [8]. In the case, we presented the patient who had numerous hospital admissions with pneumonia and left-sided pleural effusion before the diagnosis of PPF was reached.

Computed tomography scanning is useful in assessing the site and dimensions of the pleural effusion, while informing of evidence of pancreatitis and/or pancreatic pseudocysts. However, MRCP is reported to be more sensitive in assessing the presence and morphology of fistulous tracts [9]. Endoscopic retrograde cholangiopancreatography is diagnostic and therapeutic in cases of PPF [10]. An analysis of the pleural fluid will reveal significantly raised levels of amylase and lipase and together with the history and imaging will confirm the presence of a PPF.

Confirmed cases should be managed by a multidisciplinary team in a tertiary unit. There is a paucity of evidence in the literature for the optimal management strategy of pancreaticopleural fistulae and guidance is derived mainly from small case series [1, 2, 11]. The aim of treatment is to drain the pleural effusion which provides rapid pulmonary symptomatic relief and to reduce stimulation of the pancreatic exocrine secretions. This is achieved by thoracocentesis and/or tube thoracostomy and the administration of a somatostatin analogue such as octreotide for up to 6 months [4]. Reports have demonstrated that octreotide significantly reduces fistula output and decreases the time to fistula closure [11].

Endoscopic retrograde cholangiopancreatography with endoscopic pancreatic stenting is an effective and therapeutic option for fistulas present in the head and body of the pancreas. Stenting using sizes 5 or 7 Fr stents bridges areas of pancreatic ductal disruption and helps close the fistula rapidly and decrease the pancreatic ductal pressure [10]. Together with somatostatin analogues it can shorten the duration of hospital stay [10].

Surgery is indicated when conservative and endoscopic treatment fails or where obstruction of pancreatic duct cannot be relieved endoscopically. Surgical treatment includes pancreatic resection or enteropancreatic anastomosis in the form of cystogastrostomy or cyst-jejunostomy to the site of pancreatic duct leakage or to the pseudocyst [6, 11]. Although it is curative in 80–90% of cases, it carries a mortality rate of up to 10% [6, 11]. While ERCP is a less invasive alternative to surgery, a significant proportion of patients undergoing ERCP still ultimately require surgical intervention [4].

## 4. Conclusion

Pancreaticopleural fistulae are rare but must be considered in the setting of recurrent pleural effusions and coexisting pancreatitis. The diagnosis requires a high index of suspicion and the recommended treatment options include octreotide and thoracentesis and ERCP plus/minus endoscopic pancreatic stent placement. Surgery carries a significant mortality risk and is reserved for those who do not respond to medical management or develop complications.
